# Dissection versus prosection by anatomical region: Evaluating a regional group dissection curriculum in medical education

**DOI:** 10.1002/ase.70303

**Published:** 2026-07-02

**Authors:** Andrew J. Gaetano, Jason Howard, Luke Ritter, Maha Khan, Nicolas Jozefowski, Eva L. Murdoch

**Affiliations:** ^1^ Stritch School of Medicine Loyola University Chicago Maywood Illinois USA; ^2^ Department of Orthopaedic Surgery and Rehabilitation Loyola University Health System Maywood Illinois USA; ^3^ Department of Medical Education Loyola University Chicago Stritch School of Medicine Maywood Illinois USA

**Keywords:** anatomy, dissection, medical education, medical student, prosection

## Abstract

Gross anatomy education has undergone significant changes due to evolving curricular demands, leading to reduced hands‐on dissection opportunities in many medical schools. The purpose of this study was to compare the academic performance of medical student dissectors and those assigned to prosection‐based learning across anatomical regions. Dissectors performed hands‐on dissection of their assigned anatomical region, whereas the prosection group learned the same material using prosected specimens during laboratory sessions. The secondary aim was to evaluate student perceptions of the regional dissection curriculum and the educational value of dissection. In fall 2023, medical students at a single institution participated in a regional group dissection curriculum where each student was randomly assigned to dissect one anatomical region while studying all others using prosected specimens. Academic performance was assessed by comparing internally written, computer‐based multiple‐choice examinations between dissectors and prosection groups for each region using Welch's t‐tests. Additionally, student perceptions were evaluated using a post‐course survey. Examination performance was comparable between dissectors and prosection groups across most body regions, except for the anterior lower extremity, where dissectors scored significantly higher (87.91% vs. 83.15%; *p* = 0.020). Survey results indicated strong student support for the dissection experience, with over 75% agreeing it was valuable and nearly 87% reporting enhanced understanding and exam preparedness. While dissection and prosection yielded comparable examination performance across most anatomical regions, students strongly valued the dissection experience, highlighting the importance of balancing educational effectiveness, student experience, and logistical feasibility in curricular design.

## INTRODUCTION

Expanding curricular demands in medical education have led to significant changes such as reduced course durations, particularly during the preclinical years.^1^, ^2^, ^3^ While a strong foundation in human anatomy remains central to medical training, the time allocated to gross anatomy education has dramatically declined.^4^, ^5^ The average time dedicated to anatomical studies has decreased from over 800 h in 1909 to 147 h in 2004, and further declined to just 129 h in 2017.^6^, ^7^, ^8^ This trend was exacerbated by the COVID‐19 pandemic, during which many anatomy course directors reported reductions in total course time and limited opportunities for hands‐on learning.^9^

As medical education evolves, innovative teaching methodologies are necessary to adapt to changing learning environments while maintaining instructional effectiveness. Many medical schools have adopted a multimodal approach incorporating various instructional methods such as prosected specimens, computer‐aided learning, virtual reality, and advanced radiological imaging.^10^, ^11^, ^12^ Although the use of prosected specimens is not new, it is still debated whether the use of the specimens as an alternative to dissection provides comparable or superior learning outcomes. A meta‐analysis of 3035 studies by Wilson et al. found no significant difference in student performance across various anatomy teaching modalities, including prosection, models, and other hybrid approaches.^3^ Similarly, Gonsalvez et al. demonstrated that practical exam scores were comparable between students who attended dissection versus prosection lab workshops, with improved scores correlating more strongly with the total number of lab sessions attended, suggesting that increased exposure rather than the specific teaching modality plays a more significant role in student success.^13^ Additionally, a randomized trial by Koh et al. found no difference in student performance between dissection and prosection.^14^ However, these studies primarily evaluated dissection versus prosection at a course‐wide level, overlooking the potential variations in complexity among different anatomical regions.

The purpose of this study was to compare the academic performance of medical student dissectors and those assigned to prosection‐based learning across different anatomical regions. Dissector students performed hands‐on dissection of their assigned anatomical region, whereas the prosection groups learned the same material using prosected specimens during scheduled laboratory sessions. It was hypothesized that dissectors would outperform prosection groups across all regions. The secondary aim was to evaluate student perceptions of the regional dissection curriculum and the educational value of dissection. It was hypothesized that the majority of students would report favorable opinions regarding both the curriculum and the role of dissection in anatomy education.

## METHODS

This was a retrospective study performed after receiving exemption from our institutional review board.

### Curricular overview

In the 2023–2024 academic year, the institution's anatomy curriculum was structured into five units. This was a 10‐week course required for all first‐year medical students in the fall semester. Each day, students viewed two or three pre‐recorded 60‐min lectures and attended a 60‐min laboratory session featuring donor specimens prosected by the course faculty. In the 60‐min sessions, students were provided a lab guide that corresponded with the previous day's lecture and self‐explored the prosected specimens. Three faculty instructors were present at each session to assist students.

There were 11 regional group dissections including the forearm and hand, brachial plexus, posterior lower extremity, anterior lower extremity, abdomen, thorax, back with laminectomy, back with the suboccipital triangle, pharynx and larynx, superficial and deep face, and the triangles of the neck. A detailed list of the required muscles, vessels, nerves, and other structures for each dissection is provided in Supplement S1. At the beginning of the course, the students were randomly assigned to groups of 6–8 individuals to perform a regional group dissection during one designated unit of the course. Some groups performed the same dissections, and the number of students who dissected a given anatomical region can be found in Table 2. The regional group dissections were conducted in the weeks leading up to the exam covering the respective anatomical region, and the dissections required an estimated 25–30 h to complete. One instructor was present during dissection group meetings to assist the students.

At the end of each unit, all students, both dissectors and those assigned to prosection‐based learning, were formally assessed through examinations on the dissected regions. The examinations were multiple choice and were conducted via on‐campus computers. Many of the questions included images of prosections done by the course instructors and included images of donors used during the lab sessions. All exams were designed by the same instructors and contained the same balance of lower‐ and higher‐order questions.

### Data collection and analysis

Student examination scores were compiled by the research team. Scores for each anatomical region were analyzed separately for students taking the exam either assigned to the dissector group or the prosection group. The average examination scores for each group were calculated by region and compared using Welch's *t*‐test, or unequal variances *t*‐test, given the unequal group sizes and potential for variance heterogeneity. A linear mixed‐effects model was used to assess the effect of dissection on exam scores with dissection and exam number used as fixed effects and an intercept to account for repeated measurements and variability between students. All statistical analyses were conducted using IBM SPSS Statistics Version 29.0.2.0 (20).

To evaluate student perceptions of the regional group dissection curriculum and the educational value of dissection, the end‐of‐course evaluation was analyzed. This was a survey routinely administered at this institution, which included specific questions used to address the students' perceived value of regional group dissection, its impact on anatomical understanding, and its usefulness in examination preparation (Appendix A). Because the data were de‐identified and collected as part of normal educational operations, individual informed consent was not required under the institutional review board exemption. Students rated their experience using a 5‐point Likert scale. The survey was intentionally designed to evaluate student perceptions of the dissection component of the curriculum rather than to compare dissection and prosection as instructional modalities. The frequency distribution of responses was analyzed by calculating the percentage of participants selecting each response option.

## RESULTS

### Student demographics

All demographic data are summarized in Table 1. A total of 172 students participated in the anatomy course at this institution in the fall of 2023, with demographic data available for 170 students. Of these, 56 (32.9%) were men, 113 (66.5%) were women, and 1 (0.6%) preferred not to disclose their gender. A total of 126 (74.1%) students had a Bachelor of Science degree, 37 (21.8%) had a Bachelor of Arts degree, and 49 (28.8%) students had an additional graduate‐level degree. The average cumulative undergraduate grade point average (GPA) was 3.70 (SD = 0.22), and the average science GPA was 3.61 (SD = 0.29). The average Medical College Admission Test (MCAT) score was 509 (SD not available), with the most recent average MCAT score being 511 (SD = 4.63).

**TABLE 1:** Demographic data.

Characteristics	Total *n* = 170
	*N* (% or SD)
Gender	
Men	56 (32.9)
Women	113 (66.5)
Prefer not to say	1 (0.6)
Age	
<23	28 (16.5)
23–26	123 (72.4)
27–30	15 (8.8)
>30	4 (2.4)
Race	
White/Caucasian	84 (49.4)
Black/African American	5 (2.9)
East Asia/Pacific Islander/South Asian	51 (30.0)
Hispanic	14 (8.2)
Prefer not to say	16 (9.4)
Degrees	
Associate	7 (4.1)
BS	126 (74.1)
BA	37 (21.8)
Graduate	49 (28.8)
MS	28 (16.5)
MA	10 (5.9)
MPH	10 (5.9)
Other	1 (0.6)
GPA	
Cumulative	3.70 (0.22)
Science	3.61 (0.29)
Other	3.82 (0.18)
MCAT	
All average	509 (N/A)
Most recent average	511 (4.63)

Abbreviations: BA, Bachelor of Arts; BS, Bachelor of Science; GPA, grade point average; MA, Master of Arts; MCAT, Medical College Admission Test; MPH, Master of Public Health; MS, Master of Science; SD, standard deviation.

### Examination performance

Across all anatomical regions, the average examination scores for dissectors and students assigned to prosection were 82.56% and 83.04%, respectively, with no statistically significant difference between the groups (*p* = 0.631) (Table 2). A significant difference in average examination scores was observed only in the anterior lower extremity, where dissectors scored 87.91% and the prosection group scored 83.15% (*p* = 0.020).

**TABLE 2:** Examination scores by anatomical region.

Region	Dissectors		Prosection group		*p*
	*n*	Average % (SD)	*n*	Average % (SD)	
All regions combined	172	82.56 (10.39)	172	83.04 (7.94)	0.631
Upper extremity	15	75.89 (14.34)	157	79.84 (11.59)	0.316
Forearm and hand	8	76.33 (13.11)	164	79.65 (11.82)	0.503
Brachial plexus	7	75.38 (16.70)	165	79.67 (11.65)	0.525
Lower extremity	50	85.83 (9.86)	122	82.95 (9.73)	0.084
Posterior lower extremity	27	84.06 (10.74)	145	83.73 (9.69)	0.883
Anterior lower extremity	23	87.91 (8.48)	149	83.15 (9.89)	**0.020**
Ventral body cavity	30	78.37 (12.00)	142	79.73 (11.86)	0.575
Abdomen	15	78.58 (14.57)	157	79.58 (11.62)	0.780
Thorax	15	78.16 (9.26)	157	79.62 (12.09)	0.578
Back	42	83.53 (8.74)	130	83.70 (9.17)	0.914
Back with laminectomy	28	84.26 (8.91)	144	83.54 (9.09)	0.699
Back with suboccipital triangle	14	82.07 (8.52)	158	83.80 (9.10)	0.480
Head and neck	35	83.18 (7.11)	137	85.67 (6.87)	0.068
Pharynx and larynx	15	82.07 (7.96)	157	85.46 (6.82)	0.130
Superficial and deep face	13	84.72 (5.83)	159	85.20 (7.07)	0.783
Triangles of the neck	7	82.70 (7.87)	165	85.27 (6.93)	0.426

*Note*: Bolded text represents statistical significance at an alpha level of 0.05.

Abbreviation: SD, standard deviation.

Given variation between students and differences in mean scores for each exam, a linear mixed‐effects model was used to assess whether the effect of dissection on exam scores was statistically significant when controlling for exam variability and repeated student measurements. The following model was used: *Exam score ~ Dissection + Exam Number + (1 | Student)*. The coefficient for dissection was not statistically significant (*p* = 0.447). Given the class mean was significantly different between exam 2 and 4, the original model was compared to a second model using the interaction between exam and dissection variables: Exam score ~ Dissection * Exam Number + (1 | Student). The results of this test were not statistically significant (*p* = 0.35).

### Student perceptions

All students completed the post‐course survey, and responses are summarized in Figure 1. The majority of students (75.1%) agreed or strongly agreed that regional group dissection was a valuable experience in their medical education, with an average response of 4.5 on a 5‐point scale. Additionally, 86.9% of students agreed or strongly agreed that participation in dissection enhanced their understanding of the designated region more than exposure to prosected donors (average response: 4.4). Similarly, 86.9% of students felt that their assigned dissection helped them prepare for the associated exam (average response: 4.4).

**FIGURE 1:**
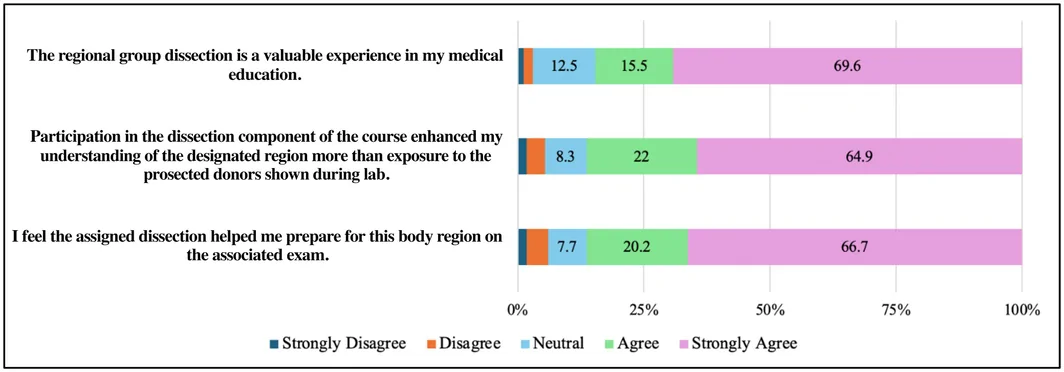
Student responses to the post‐course survey.

## DISCUSSION

The purpose of this study was to compare the academic performance of medical student dissectors and those assigned to prosection‐based learning by anatomical region. There were no significant differences in examination performance between the two groups across most anatomical regions, except for the anterior lower extremity, where dissectors performed significantly better than those assigned to prosection. Additionally, no significant impact of dissection was observed when controlling for exam difficulty, exam variation, and repeated student measurements in the linear models. These results did not support the initial hypothesis that dissectors would consistently outperform students assigned to prosection‐based learning.

Previous studies have yielded both similar and contrasting findings to those of this study. For example, Wilson et al., Gonsalvez et al., and Koh et al. showed that student performance in anatomy education did not differ significantly between dissection and prosection approaches.^3^, ^13^, ^14^ Gonsalvez et al. also emphasized that increased lab exposure was the primary factor contributing to improved outcomes. However, these studies primarily compared dissection and prosection at a course‐wide level without accounting for the complexity of specific anatomical regions.

Few studies have directly compared the educational value of dissection and prosection by anatomical region. Aziz et al. compared the practical examination scores of medical students who either dissected donors or reviewed prosected specimens.^15^ In that curriculum, students alternated regularly between dissector and non‐dissector roles, such that all students performed approximately half of the required dissections over the course. Assessment relied primarily on donor‐based, first‐order identification questions derived from all dissected regions. Although the dissection group scored higher than the prosection group in regions such as the pelvis and lower limb, the alternating structure of the curriculum with frequent dissection exposure across regions may have limited the ability to isolate the effect of hands‐on dissection versus prosection for specific anatomical regions. Coker et al. assessed the impact of dissection‐based versus prosection‐based curricula on student performance by comparing two consecutive medical student cohorts in which head and neck anatomy was taught primarily through either student‐led dissection or faculty‐prepared prosections.^16^ In that study, students were not divided into concurrent dissector and prosection groups within the same course; rather, entire class cohorts experienced a single dominant instructional modality. Both cohorts received equivalent laboratory contact hours, were responsible for the same structure lists, and were assessed using identical donor‐based practical examinations that emphasized first‐order identification questions, with a smaller proportion of second‐order functional questions. Because exposure time, content scope, and assessment format were held constant, the authors were able to isolate modality‐level differences but could not evaluate region‐specific effects within a hybrid curriculum. This contrasts with our study design, in which students dissected a single assigned region while learning all other regions via prosected donor specimens, allowing for direct comparison of dissection versus prosection at the anatomical‐region level within the same cohort. Similarly, Williams et al. compared a dissection‐focused curriculum with a prosection‐focused approach when teaching the hand and foot regions.^17^ In this curriculum, students were not assigned to permanent dissector or prosection‐based learning roles. Instead, an alternating dissection model was used in which students dissected approximately half of the course content and learned the remaining material through peer‐teaching and interactions with prosected specimens. Both cohorts received substantial laboratory contact time and were responsible for the same comprehensive structure lists. Student performance was assessed using donor‐based practical examinations that emphasized first‐order structure identification, with a smaller proportion of second‐order functional questions. The authors found that students who learned the hand and foot through prosected specimens outperformed those who dissected these regions, a finding they attributed to improved preservation and visualization of small, intricate structures that are traditionally difficult for novice learners to dissect. Because all students in this model experienced both dissection and prosection across different regions, the study could not fully isolate the impact of hands‐on dissection on performance within a single anatomical region, in contrast to the region‐specific assignment used in our curriculum. Overall, our findings are consistent with prior work suggesting that within hybrid curricula, dissection does not uniformly improve examination performance compared with prosection.

In the present study, there was notable variation in examination performance across regions, raising the possibility that region‐specific benefits may emerge depending on anatomical complexity. Although the linear model did not show an interaction between exam and dissection, it may be hypothesized that some anatomic regions are particularly suited to hands‐on exploration, whereas others may be adequately mastered through prosection. This is supported by prior research that suggests regions with intricate fascial planes, layered musculature, or challenging neurovascular arrangements may benefit more from hands‐on dissection, whereas more linear or superficial regions may be adequately learned through prosection.^15^, ^16^, ^17^ The anterior lower extremity, the only region in which dissectors significantly outperformed the prosection group in the current study, includes several such elements including the femoral triangle, profunda branches, and compartmental organization, which may lend themselves more effectively to active exploration than static review. This may partially explain why there was an observed performance advantage in this region but not in others. Conversely, the absence of significant performance differences in other regions suggests that prosection‐based exposure may provide sufficient visual and conceptual understanding for many anatomical areas. Because all students had daily access to prosected donors during structured laboratory sessions, the baseline level of exposure was high across the cohort. Under these circumstances, the marginal benefit of additional hands‐on dissection may be attenuated, particularly in regions with more superficial structures or clearer anatomical boundaries. This aligns with prior work indicating that overall exposure time, rather than dissection modality, is the strongest predictor of performance.^13^

The secondary aim of this study was to assess student perceptions of the regional dissection curriculum and the educational value of dissection. Consistent with our hypothesis, the majority of students viewed regional group dissection as a valuable component of their medical education, reported enhanced understanding of their assigned anatomical region compared with exposure to prosected donors alone, and felt that participation in dissection improved preparation for the corresponding examination.

Importantly, these findings suggest that the educational value of dissection extends beyond its measurable impact on examination performance. Students frequently described dissection as contributing to spatial reasoning, teamwork, communication, and professional identity formation, competencies not captured by written or practical examinations. Prior studies similarly demonstrate that dissection fosters emotional resilience, respect for donors, anatomical curiosity, and collaborative problem‐solving.^18^, ^19^ Dissabandara et al. reported more favorable student attitudes toward dissection‐based learning, particularly among students who regularly attended laboratory sessions.^18^ Similarly, Cho et al. found that medical students consistently preferred hands‐on dissection and ranked anatomy among the most important preclinical subjects.^19^ Taken together, these data suggest that while dissection may not consistently confer an advantage in examination performance, it remains a highly valued educational experience that contributes meaningfully to students' overall development. These findings support a nuanced approach to anatomy curriculum design that balances efficiency with the cognitive, professional, and experiential benefits of dissection.

In redesigning medical school curricula, particularly in anatomy traditionally taught through dissection, it is essential to balance efficiency with educational value. While dissection may not always be the most time‐efficient method, this study highlights its significant value and the impact it has on students' learning experiences. Importantly, anatomy teaching methods vary widely across the world, shaped in part by differences in body donor availability and the legal, cultural, and ethical frameworks governing donor donation.^20^ In the United States, reliance on willed body donation combined with curricular and logistical constraints has contributed to the adoption of prosection‐heavy or hybrid instructional models at many institutions. In contrast, many European institutions, particularly in Western Europe where body donation programs are well established, routinely integrate student dissection with prosection, often supplementing hands‐on dissection with faculty‐prepared prosected specimens. This underscores that the question is not necessarily which method is superior, but how best to integrate the strengths of each to maximize educational value. Before modifying or eliminating components of medical curricula, their effects should be carefully evaluated and student perceptions considered to ensure that future generations of physicians receive the most comprehensive, effective, and meaningful education possible.

This study has limitations. First, the data were collected from a single institution during one academic year, which may limit the generalizability of the findings. Second, due to time constraints, some anatomical regions, such as the inguinal region and pelvis, were not included in the analysis. Third, individual variations in students' educational experiences could not be controlled for, and it could not be ensured that all students assigned to a given dissection actively participated. Fourth, the study design did not include a pre‐intervention assessment to establish individual student baselines, as participation in regional dissection was randomized and embedded within the broader curriculum, making region‐specific pre‐testing logistically challenging and potentially disruptive to the learning flow. Fifth, the qualitative data collected focused exclusively on student perceptions of the regional group dissection experience. Because the survey was developed specifically to evaluate the dissection curricular component, it did not include complementary questions addressing the perceived utility of prosection, creating an asymmetry in qualitative data which limited the ability to draw direct comparisons between the two modalities. Future studies incorporating balanced qualitative items for both dissection and prosection would allow for a more comprehensive assessment of student perspectives.

## CONCLUSION

While dissection and prosection yielded comparable examination performance across most anatomical regions, students strongly valued the dissection experience, highlighting the importance of balancing educational effectiveness, student experience, and logistical feasibility in curriculum design.

## AUTHOR CONTRIBUTIONS

**Andrew J. Gaetano:** Conceptualization; investigation; writing – original draft; methodology; validation; visualization; writing – review and editing; formal analysis; project administration; supervision; resources. **Eva L. Murdoch:** Supervision; resources; project administration; writing – original draft; writing – review and editing. **Nicolas Jozefowski:** Investigation; writing – original draft; writing – review and editing. **Luke Ritter:** Writing – original draft; investigation; visualization; writing – review and editing. **Maha Khan:** Investigation; writing – original draft; writing – review and editing. **Jason Howard:** Writing – original draft; investigation; methodology; visualization; writing – review and editing.

## CONFLICT OF INTEREST STATEMENT

The authors declare no conflicts of interest.

## Supporting information


Data S1.

## Data Availability

The data that support the findings of this study are available on request from the corresponding author. The data are not publicly available due to privacy or ethical restrictions.

## APPENDIX A POST‐COURSE SURVEY QUESTIONS

**Table:**

On a scale from 0 (strongly disagree) to 5 (strongly agree)…
The regional group dissection is a valuable experience in my medical education Participation in the dissection component of the course enhanced my understanding of the designated region more than exposure to the prosected donors shown during the lab I feel the assigned dissection helped me prepare for this body region on the associated exam

## References

[1] Dinsmore CE , Daugherty S , Zeitz HJ . Teaching and learning gross anatomy: dissection, prosection, or “both of the above?”. Clin Anat. 1999;12(2):110–114. 10.1002/(SICI)1098-2353(1999)12:2<110::AID-CA5>3.0.CO;2-3 10089036

[2] Sugand K , Abrahams P , Khurana A . The anatomy of anatomy: a review for its modernization. Anat Sci Educ. 2010;3(2):83–93. 10.1002/ase.139 20205265

[3] Wilson AB , Miller CH , Klein BA , Taylor MA , Goodwin M , Boyle EK , et al. A meta‐analysis of anatomy laboratory pedagogies. Clin Anat. 2018;31(1):122–133. 10.1002/ca.22934 28612403

[4] Elizondo‐Omaña RE , Guzmán‐López S , García‐Rodríguez ML . Dissection as a teaching tool: past, present, and future. Anat Rec B New Anat. 2005;285(1):11–15. 10.1002/ar.b.20070 16032753

[5] Iwanaga J , Loukas M , Dumont AS , Tubbs RS . A review of anatomy education during and after the COVID‐19 pandemic: revisiting traditional and modern methods to achieve future innovation. Clin Anat. 2021;34(1):108–114. 10.1002/ca.23655 PMC7404762 32681805

[6] Bardeen CR . Report of the sub‐committee on anatomy to the council on medical education of the American Medical Association. Anat Rec. 1909;3:415–439. 10.1002/ar.1090030705

[7] Drake RL , McBride JM , Pawlina W . An update on the status of anatomical sciences education in United States medical schools. Anat Sci Educ. 2014;7(4):321–325. 10.1002/ase.1468 24895314

[8] McBride JM , Drake RL . National survey on anatomical sciences in medical education. Anat Sci Educ. 2018;11(1):7–14. 10.1002/ase.1760 29265741

[9] Shin M , Prasad A , Sabo G , Macnow ASR , Sheth NP , Cross MB , et al. Anatomy education in US medical schools: before, during, and beyond COVID‐19. BMC Med Educ. 2022;22(1):103. 10.1186/s12909-022-03177-1 PMC8851737 35172819

[10] Azer SA , Eizenberg N . Do we need dissection in an integrated problem‐based learning medical course? Perceptions of first‐ and second‐year students. Surg Radiol Anat. 2007;29(2):173–180. 10.1007/s00276-007-0180-x 17318286

[11] Gunderman RB , Wilson PK . Viewpoint: exploring the human interior: the roles of cadaver dissection and radiologic imaging in teaching anatomy. Acad Med. 2005;80(8):745–749. 10.1097/00001888-200508000-00008 16043529

[12] Uruthiralingam U , Rea PM . Augmented and virtual reality in anatomical education—a systematic review. Adv Exp Med Biol. 2020;1235:89–101. 10.1007/978-3-030-37639-0_5 32488637

[13] Gonsalvez DG , Ovens M , Ivanusic J . Does attendance at anatomy practical classes correlate with assessment outcome? A retrospective study of a large cohort of undergraduate anatomy students. BMC Med Educ. 2015;15:231. 10.1186/s12909-015-0515-y PMC4688956 26698839

[14] Koh ZJ , Yeow M , Srinivasan DK , Ng YK , Ponnamperuma GG , Chong CS . A randomized trial comparing cadaveric dissection and examination of prosections as applied surgical anatomy teaching pedagogies. Anat Sci Educ. 2023;16(1):57–70. 10.1002/ase.2166 34968002

[15] Aziz M , Kernick ET , Beck Dallaghan GL , Gilliland KO . Dissection versus prosection: a comparison of laboratory practical examinations. Med Sci Educ. 2019;30(1):47–51. 10.1007/s40670-019-00839-6 PMC8368320 34457635

[16] Coker C , Rogers RS , Freed B , Steele R , Kinde MN , Danilova G , et al. Battle of the sections: student outcomes and course feedback support combined prosection and dissection laboratory formats to maximize student success. Anat Sci Educ. 2024;17(5):1012–1025. 10.1002/ase.2420 38570916

[17] Williams SR , Thompson KL , Notebaert AJ , Sinning AR . Prosection or dissection: which is best for teaching the anatomy of the hand and foot? Anat Sci Educ. 2019;12(2):173–180. 10.1002/ase.1808 30118189

[18] Dissabandara LO , Nirthanan SN , Khoo TK , Tedman R . Role of cadaveric dissections in modern medical curricula: a study on student perceptions. Anat Cell Biol. 2015;48(3):205–212. 10.5115/acb.2015.48.3.205 PMC4582164 26417481

[19] Cho MJ , Hwang YI . Students' perception of anatomy education at a Korean medical college with respect to time and contents. Anat Cell Biol. 2013;46(2):157–162. 10.5115/acb.2013.46.2.157 PMC3713280 23869263

[20] Habicht JL , Kiessling C , Winkelmann A . Bodies for anatomy education in medical schools: an overview of the sources of cadavers worldwide. Acad Med. 2018;93(9):1293–1300. 10.1097/ACM.0000000000002227 PMC6112846 29561275

