# Supplementary material for: Dissection versus prosection by anatomical region: Evaluating a regional group dissection curriculum in medical education
Source: Anat Sci Educ. 2026 Jul 2;19(11):1881–7. doi: 10.1002/ase.70303 (PMC13736530; doi:10.1002/ase.70303)
Supplement: Supplementary file 1 [file ASE-19-1881-s001.docx]

**Supplement 1**. Detailed list of the muscles, vessels, nerves, and other anatomy that students were required to locate during their group dissections.

| **General region** | **Localized regions** | **Structures to find** |
| --- | --- | --- |
| **Upper extremity** | Forearm and hand | *Muscles*:  Pronator teres m.  Flexor carpi radialis longus  Palmaris longus  Flexor carpi ulnaris  Flexor digitorum superficialis  Flexor pollicis longus  Flexor digitorum profundus  Pronator quadratus  Brachioradialis  Flexor pollicis brevis  Abductor pollicis brevis  Opponens pollicis  Flexor digiti minimi  Abductor digiti minimi  opponens digiti minimi  Lumbricals  *Nerves*:  Median  Anterior interosseous  Radial  Superficial branch of radial  Deep branch of radial  Ulnar  Superficial branch of ulnar  Deep branch of ulnar  Recurrent branch of median  *Vessels (arteries and veins)*:  Brachial a.  Radial a.  Ulnar a.  Common interosseous a.  Anterior interosseous a.  Posterior interosseous a.  Superficial palmar arch  Superior branch of ulnar a.  Other:  Flexor retinaculum  Palmar aponeurosis |
|  | Brachial plexus | *Muscles*:  Pectoralis major  Pectoralis minor  Subscapularis  Serratus anterior  *Vessels (arteries and veins):*  Axillary a.  Supreme thoracic a.  Thoracoacromial a.  Lateral thoracic a.  Anterior circumflex humeral a.  Posterior circumflex humeral a.  Subscapular a.  Circumflex scapular a.  Thoracodorsal a.  Pectoral a.  *Nerves*:  Medial, lateral, and posterior cords  Long thoracic  Medial pectoral  Lateral pectoral n.  Medial antebrachial cutaneous n.  Medial brachial cutaneous n.  Ulnar n.  Medial n.  Musculocutaneous n.  Axillary n.  Radial n.  Thoracodorsal n.  Upper scapular n.  Lower scapular n. |
| **Lower extremity** | Posterior lower extremity | *Muscles*:  Gluteal region:  Gluteus maximus m.  Gluteus medius m.  Gluteus minimus m.  Piriformis m.  Obturator internus m. and tendon  Superior gemellus m.  Inferior gemellus m.  Quadratus femoris m.  Tensor fasciae lata m.  Posterior thigh region:  Long head of biceps femoris m.  Short head of biceps femoris m.  Semitendinosus m.  Semimembranosus m.  Posterior leg region:  Gastrocnemius m.  Soleus m.  Plantaris m.  Calcaneal tendon  Popliteus m.  Flexor digitorum longus m.  Flexor hallucis longus m.  Tibialis posterior m.  *Nerves*:  Sciatic n.  Tibial n.  Common fibular n.  Superior gluteal n.  Inferior gluteal n.  Posterior femoral cutaneous n.  Nerve to obturator internus  Pudendal n.  Saphenous n.  Sural n.  Common fibular n.  *Vessels (arteries and veins):*  Superior gluteal a.  Inferior gluteal a.  Internal pudendal a.  Popliteal a.  Anterior tibial a.  Posterior tibial a.  Fibular a.  *Other*:  Sacrotuberous ligament  Sacrospinous ligament |
|  | Anterior lower extremity | *Anterior compartment muscles of thigh*:  Iliopsoas m.  Sartorius m.  Quadriceps femoris m. (rectus femoris m., vastus lateralis m., vastus medialis m., vastus intermedius m.)  Quadriceps tendon  Patellar ligament  *Medial compartment muscles of the thigh:*  Adductor longus m.  Adductor brevis m.  Adductor magnus m.  Pectineus m.  Gracilis m.  Pes anserinus insertion  *Nerves of thigh*:  Femoral n.  Lateral femoral cutaneous n.  Saphenous n.  Obturator n.  *Vessels of the thigh (arteries and veins):*  Great saphenous v.  Femoral a.  Femoral v.  Deep femoral a. (perforating arteries, lateral circumflex femoral a., medial circumflex femoral a.)  *Muscles of the anterior compartment of the leg:*  Tibialis anterior m.  Extensor hallucis longus m.  Extensor digitorum longus m.  Fibularis tertius m.  *Lateral compartment muscles of the leg*:  Fibularis longus m.  Fibularis brevis m.  *Dorsal muscles of the foot*:  Extensor pollicis brevis m.  Extensor digitorum brevis m.  *Nerves of the leg*:  Saphenous n.  Common fibular n. (deep fibular n., superficial fibular n.)  *Vessels of the leg (arteries and veins):*  Anterior tibial a. (dorsalis pedis a. – arcuate a. and deep plantar a.)  *Other*:  Adductor hiatus |
| **Ventral body cavity** | Abdomen | *Muscles*:  Psoas major m.  Iliacus m.  Quadratus lumborum m.  Diaphragm  *Organs*:  Liver  Gallbladder (cystic duct, common hepatic duct, common bile duct)  Stomach (greater omentum)  Pancreas  Duodenum, jejunum, ileum  Cecum, appendix, colon  Spleen  Kidney and ureter  *Vessels (arteries and veins):*  Abdominal aorta (celiac trunk – left gastric a., splenic a., common hepatic as., proper hepatic as. With left and right hepatic a.; superior mesenteric a.; inferior mesenteric a.; renal a.; common iliac a.)  Inferior vena cava  Hepatic portal v. (splenic v.; inferior mesenteric v.; superior mesenteric v.)  Renal v.  Gonadal v.  Common iliac v.  *Nerves*:  Greater splanchnic n.  Lateral femoral cutaneous n.  Femoral n.  Obturator n. |
|  | Thorax | *Muscles*:  External intercostal m.  Internal intercostal m.  Transverse thoracis m.  Diaphragm m.  *Nerves*:  Intercostal ns.  Phrenic n.  Vagus n. (X); right and left recurrent laryngeal ns.  *Vessels (arteries and veins):*  Ascending aorta  Arch of aorta (brachiocephalic trunk – right subclavian a. and right common carotid a.; left common carotid a.; left subclavian a.)  Right and left subclavian as. (Vertebral a; internal thoracic a. – anterior intercostal a.; thyrocervical trunk)  Thoracic aorta - descending aorta (posterior intercostal a.)  Pulmonary trunk (Right and left pulmonary as.)  Inferior vena cava  Superior vena cava (Right and left brachiocephalic vs.)  Subclavian v. (internal jugular v.; internal thoracic v. – anterior intercostal v.)  Azygous v. (posterior intercostal v.)  *Other*:  Ligamentum arteriosum  Trachea  Esophagus  Thoracic duct |
| **Back** | Back with laminectomy | *Superficial back muscles:*  Trapezius  Latissimus dorsi  Levator scapulae  Rhomboid minor  Rhomboid major  Serratus posterior superior  Serratus posterior inferior  *Deep back muscles:*  Splenius capitis  Semispinalis capitis  Erector spinae (iliocostalis, longissimus, spinalis)  *Posterior shoulder muscles:*  Deltoid  Long head of triceps brachii  Teres major  Teres minor  Supraspinatus  Infraspinatus  *Vessels (arteries and veins):*  Transverse cervical a. (superficial branch)  Thoracodorsal a.  Dorsal scapular a.  Posterior circumflex humeral a.  Suprascapular a.  Circumflex scapular a.  *Nerves:*  Greater occipital  Spinal accessory (XI)  Dorsal scapular  Thoracodorsal  Axillary  Suprascapular  Spinal cord  Conus medullaris  Dorsal root  Dorsal root ganglia  Ventral root  Ventral ramus  Spinal nerve  Cauda equina  *Other*:  Dura mater  Interspinous ligament  Ligamentum flavum  Filum terminale  Denticulate ligaments  Quadrangular space  Triangular space |
|  | Back with suboccipital triangle | *Superficial muscles:*  Trapezius  Latissimus dorsi  Levator scapulae  Rhomboid minor  Rhomboid major  Serratus posterior superior  Serratus posterior inferior  *Deep back muscles:*  Splenius capitis  Semispinalis capitis  Erector spinae (iliocostalis, longissimus, spinalis)  *Suboccipital muscles:*  Obliquus capitis inferior  Obliquus capitis superior  Rectus capitis posterior minor  Rectus capitis posterior major  *Posterior shoulder muscles:*  Deltoid  Long head of triceps brachii  Teres major  Teres minor  Supraspinatus  Infraspinatus  *Nerves*:  Spinal accessory (XI)  Thoracodorsal  Greater occipital  Suboccipital  Axillary  Suprascapular  *Vessels (arteries and veins):*  Transverse cervical a. (superficial branch)  Thoracodorsal a.  Occipital a.  Vertebral a.  Posterior circumflex humoral a.  Circumflex scapular a.  Suprascapular a. |
| **Head and neck** | Pharynx and larynx | **Pharynx**:  *Muscles*:  Superior pharyngeal constrictor  Middle pharyngeal constrictor  Inferior pharyngeal constrictor  Stylopharyngeus  *Nerves*:  Glossopharyngeal (IX)  Spinal accessory (XI)  Hypoglossal (XII)  Sympathetic chain and superior cervical ganglion  Vagus (X) with superior laryngeal nerve  *Vessels (arteries and veins):*  Internal jugular v.  Common carotid a.  Internal carotid a.  External carotid a.  *Other*:  Torus tubarius  Epiglottis  **Larynx**:  *Muscles*:  Posterior cricoarytenoid  Transverse arytenoid  Oblique arytenoid  Cricothyroid  *Nerves*:  Superior laryngeal (external and internal laryngeal nerves)  Recurrent laryngeal  *Vessels (arteries and veins):*  Superior laryngeal a.  Inferior thyroid a.  *Other*:  Thyroid cartilage  Cricoid cartilage  Thyrohyoid membrane  Cricothyroid ligament |
|  | Superficial and deep face | **Superficial face**:  *Muscles*:  Orbicularis oculi  Orbicular oris  Frontalis  Buccinator  Zygomaticus major  Masseter  Temporalis  *Nerves*:  Facial (VII)  *Vessels (arteries and veins):*  Superficial temporal a.  Facial a.  *Other*:  Parotid gland  Stensen duct  **Infratemporal fossa**:  *Nerves*:  Auriculotemporal  Lingual  Chorda tympani  Inferior alveolar  Nerve to mylohyoid  *Vessels (arteries and veins):*  Superficial temporal a.  Maxillary a.  Middle meningeal a.  Inferior alveolar a.  Buccal a.  Infraorbital a.  Posterior superior alveolar a. |
|  | Triangles of the neck | **Posterior triangle**:  *Muscles*:  Platysma  Sternocleidomastoid  Omohyoid (inferior belly)  *Nerves*:  Accessory (XI)  Phrenic  *Vessels (arteries and veins):*  Inferior thyroid a.  Suprascapular a.  Transverse cercival a.  **Anterior triangle:**  *Muscles*:  Sternocleidomastoid  Sternohyoid  Sternothyroid  Thyrohyoid  Omohyoid (superior belly)  Hylohyoid  Stylohyoid  Digastric (anterior and posterior bellies)  *Nerves*:  Vagus (X); superior laryngeal nerve (external and internal laryngeal nerves) and recurrent laryngeal nerves  Hypoglossal (XII)  *Vessels (arteries and veins):*  Superior thyroid a.  Lingual a.  Facial a.  Common carotid a.  External carotid a.  Internal carotid a.  Internal jugular v.  *Other*:  Submandibular gland  Parotid gland  Thyroid gland |
